# TLE4 Is a Critical Mediator of Osteoblast and Runx2-Dependent Bone Development

**DOI:** 10.3389/fcell.2021.671029

**Published:** 2021-08-06

**Authors:** Thomas H. Shin, Evangelos Theodorou, Carl Holland, Rae’e Yamin, Cathleen L. Raggio, Philip F. Giampietro, David A. Sweetser

**Affiliations:** ^1^Department of Pediatrics, Center of Genomic Medicine, Divisions of Pediatric Hematology/Oncology and Medical Genetics, Massachusetts General Hospital, Harvard Medical School, Boston, MA, United States; ^2^Department of Molecular and Translational Medicine, Boston University School of Medicine, Boston, MA, United States; ^3^Department of Pediatric Orthopedics, Hospital for Special Surgery, New York, NY, United States; ^4^Department of Pediatrics, University of Illinois, Chicago, IL, United States

**Keywords:** *Tle4*, osteoblast, *Runx2*, bone mineralization, Tle4-Runx axis, bone calcification

## Abstract

Healthy bone homeostasis hinges upon a delicate balance and regulation of multiple processes that contribute to bone development and metabolism. While examining hematopoietic regulation by *Tle4*, we have uncovered a previously unappreciated role of *Tle4* on bone calcification using a novel *Tle4* null mouse model. Given the significance of osteoblasts in both hematopoiesis and bone development, this study investigated how loss of *Tle4* affects osteoblast function. We used dynamic bone formation parameters and microCT to characterize the adverse effects of *Tle4* loss on bone development. We further demonstrated loss of *Tle4* impacts expression of several key osteoblastogenic genes, including *Runx2*, *Oc*, and *Ap*, pointing toward a potential novel mechanism for *Tle4*-dependent regulation of mammalian bone development in collaboration with the RUNX family members.

## Introduction

Normal bone development is a dynamic process that depends on the balance between bone formation and bone resorption. These two processes are largely mediated by osteoblasts and osteoclasts, respectively. An imbalance of these two forces results in various bone pathologies, including osteopetrosis and osteoporosis (Cohen, 2006; Kaul et al., 2015). Osteoblasts are derived from mesenchymal cells that are triggered by Wnt signaling toward osteoblastic differentiation (Hill et al., 2005; Houschyar et al., 2019). Various factors, including Wnt, BMP signaling, and *Runx2*, have been found to play roles, not only in normal osteoblast function, but also maturation and viability (Cohen, 2006; Kozhemyakina et al., 2015). *Runx2* and *Osterix* regulate the differentiation of mesenchymal stem cells (MSCs) to osteoblastic lineages (Asada and Katayama, 2014). The loss of *Runx2*, a known interaction partner of *Tle* co-repressors, results in the absence of bone formation thought to be secondary to aberrant osteoblast differentiation in mice (Choi et al., 2001). Additionally, previous studies have shown that *Runx2* expression induces osteoblastic differentiation of mouse stromal cells (Baniwal et al., 2012). Runx2 augments mesenchymal lineage proliferation while also assisting the commitment to osteoblasts by regulating a series of signaling pathways that include Wnt, FGF and PTH, as well as *Dlx5* (Komori, 2019). Moreover, *Runx2* enhances the expression of bone matrix protein genes including *Col1a1, Spp1, Ibsp, Bglap2* and *Fin1* (Komori, 2019). *Smad* and p38 *MAPK* signaling pathways regulate *Runx2* promoting osteoblast and chondrocyte differentiation (Wu et al., 2016). Other key regulators of osteoblast differentiation include osteoproteregin, osterix, and alkaline phosphatase, and osteopontin. These factors are expressed at different stages of osteoblast differentiation and regulate osteoblast precursor fate decisions, bone metabolism, and osteoclast induction (Cohen, 2006).

The Groucho/TLE family of proteins are intimately involved in the regulation of various signaling pathways critical to cell fate and development, including receptor tyrosine kinase/Ras/MAPK, Notch, and Wnt signaling (Zhang and Dressler, 2013; Chodaparambil et al., 2014). The Groucho/TLE family have been extensively studied as corepressors of various binding partners, including the RUNX/AML family through the C-terminal VWRPY Groucho recruitment motif (Levanon et al., 1998; Chen and Courey, 2000). In leukemia, we have shown *t*(8;21) leukemic cell viability and growth are sensitive to TLE4 levels and that loss of the TLE homolog in zebrafish, Gro3, cooperates with AML1-ETO to create a myeloid leukemia phenotype (AML) (Dayyani et al., 2008). Having identified the tumor suppressor role of *TLE4* in myeloid leukemias, we generated a novel *Tle4* knockout mouse model to better understand its role in mammalian development (Sweetser et al., 2005; Dayyani et al., 2008; Wheat et al., 2014). In addition to various hematopoietic abnormalities, we unexpectedly found a severe bone development defect in these mice leading to severe runting and decreased bone mineralization (Wheat et al., 2014). Similar dual functions have been described for other regulators of bone development that also can function as tumor suppressor genes including FoxO members and ARF which drives bone remodeling and osteosarcoma development in mice through both p53 independent and dependent mechanisms (Rauch et al., 2010; Le et al., 2018; Ma et al., 2018; Schmitt-Ney, 2020).

Hematopoiesis and normal bone development are intimately connected (Bianco, 2011; Despars and St-Pierre, 2011). In concert with other resident bone tissue cells, osteoblasts create and protect a hospitable hematopoietic stem cell (HSC) microenvironment (Cohen, 2006; Yin and Li, 2006). Co-cultures of MSCs with various leukemic cell lines increase osteoblastic markers such as *Runx2, Osx, Opn* (Le et al., 2018). Initial studies of hematopoiesis in the bone marrow found many HSCs in close proximity to the inner bone endosteal area, which has triggered much inquiry into the specific relevance of osteoblasts in HSC maintenance and niche (Yin and Li, 2006; Garcia-Garcia et al., 2015). Osteoblasts communicate with HSCs through direct receptor-ligand interactions (e.g., Ang1/Tie2 and TPO/MPL), to support HSC adhesion and residence in the niche, including interactions between N-cadherin/β-catenin, and osteopontin/β_1_integrin (Yin and Li, 2006; Le et al., 2018). In addition, osteoblasts secrete factors including G-CSF, hepatocyte growth factor and osteopontin that regulate the pool size of the CD34^+^ progenitor population (Le et al., 2018). Osteoblasts regulate HSC migration in and outside of the bone marrow through CXCL12/CXCR4 and VCAM-1/VLA-4 (Le et al., 2018). The constitutive activation of β-catenin in osteoblasts and resultant expression of the Notch ligand Jagged-1 activates Notch signaling in HSC leading to the development of AML. This underscores the importance of normal regulation of mediators of osteoblast differentiation on normal hematopoiesis (Kode et al., 2014). *Dicer1* or *Ptpn11* deficient osteoprogenitor cells in mice display myelodysplastic syndrome and secondary acute myeloid leukemias, as well as juvenile myelomonocytic leukemia-like myeloproliferative neoplasms, respectively (Le et al., 2018). This is further demonstrated by studies that found HSC populations increased in parallel with expansion of osteoblasts due to parathyroid hormone treatment in mice (Calvi et al., 2003), while PTH activation enhances the migration of long-term repopulating HSCs (Even et al., 2021). Moreover, a previous study targeted ablation of osteoblasts in mice found that the loss of osteoblasts significantly reduced HSC and hematopoietic progenitor populations (Visnjic et al., 2004). Furthermore, animals deficient in Sca marker present bone abnormalities (Aguila and Rowe, 2005). Thus, osteoblast function intimately connects bone formation and hematopoiesis. Specifically Car/LepR^+^ CXCL12 expressing cells create a niche for HSCs cells while simultaneously give rise to osteoblasts (Galan-Diez and Kousteni, 2018). The similarity of *Tle4* null mice to *Runx2* null mice suggested the loss of *Tle4* might either impair the function or the expression of *Runx2*. To better characterize the nature of the defect in bone development and maintenance in *Tle4* null mice we have used *Tle4* null and conditional *Tle4* knockout mice and performed assays of osteoblast function and development in bone stromal cultures and mesenchymal bone marrow cell lines.

## Materials and Methods

### Generation of *Tle4* Null and Conditional *Tle4* Knockout Mice

For these experiments we used *Tle4* null (T4KO) and *Tle4* conditional knockout mice generated in our laboratory as previously described (Wheat et al., 2014). Briefly, conditional mice were constructed by targeting LoxP sites to flank exon 2 of *Tle4 via* homologous recombination using the 129S6/SvEvTac ES cell line (T4F). To generate T4KO, resultant mice were crossed with β-actin:Cre mice (gift of Dr. Susan Dymecki) to delete exon 2 in all tissues. Heterozygote mice were backcrossed to C57BL/6 background for over six generations and interbred to generate *Tle4* null mice. For conditional knockout of *Tle4*, homozygous T4F mice containing *Mx1*-Cre (T4F cre) were used. Excision of *Tle4* exon 2 was induced with three intraperitoneal injections of 15 mg/kg polyinosinic-polycytidylic acid (pIpC; Sigma) separated by 48 h. pIpC treatment induces interferon-γ signaling with activation of Cre expression and subsequent Cre recombinase excision of exon 2 of *Tle4.* This is predicted to cause a frameshift resulting in a premature stop codon and non-functional truncated *Tle4* protein (Wheat et al., 2014). Demonstration of T4F knockout efficiency by pIpC was performed by PCR using primers mT4WTvFlpR 5′- GGAGACTTGGAAAACGCTGA-3′, mT4PcreF 5′- CAAAGGGCCCCAGAATCTT-3′and mT4PcreR 5′- CGACCGACTTGTAGCCATTT-3′. Mice were housed in a specific pathogen-free environment with a 12-h light/dark cycle, 30–70% relative humidity and approximately 70°F ambient temperature, in groups not surpassing four adult animals. Mice had *ad libitum* access to tap water and standard rodent chow (Prolab^®^ RMH 3500, Scotts Distributing, Hudson, NH, United States). For analysis mice were euthanized by inhalation of 100% CO2. This study was carried out in strict accordance with recommendations in the Guide for the Care and Use of Laboratory Animals of the National Institutes of Health and approved by the Massachusetts General Hospital Institutional Animal Care and Use Committee.

### Cell Culture, shRNA Construction, and Lentiviral Infection

ST2D cells (Generous gift of Dr. Baruch Frenkel) were generated by stably transforming mouse mesenchymal ST2 cells derived from bone marrow with a doxycycline-inducible *Runx2* expression vector (Baniwal et al., 2012). ST2D cells were cultured in RPMI-1640 (Lonza, Walkersville, MD, United States) supplemented with 10% FBS (Sigma-Aldrich, St. Louis, MO, United States) and 1% penicillin/streptomycin (Invitrogen, United States). Cells were maintained at 37°C and 5% CO_2_. When indicated, cells were also cultured with 350 ng/mL doxycycline (Sigma-Aldrich, United States) or DMSO (Sigma-Aldrich, United States). Non-targeting control (scr) and Tle4-specific shRNA constructs were developed using the lentiviral vector FUGW and delivered to cells *via* lentiviral delivery as previously described (Dayyani et al., 2008). The Tle4 shRNA (shTle4) used has the following target sequence: AGTGATGACAACTTGGTGG and a control scrambled shRNA (scr) CAGTCGCCATTAGTTCCAC. Infected cells were identified by GFP fluorescence detected using FACS LSRII or GFP-selected *via* cell sorting with FACS Aria (BD, United States).

### Generation of Stromal Cultures

Stromal cultures were generated from bones of 1-week old T4WT or T4KO littermates as previously described (Mukherjee et al., 2008; Wheat et al., 2014). After harvesting femur and humeri, whole bones were crushed and plated on tissue culture plates with MEMα (Invitrogen, United States) supplemented with 20% FBS and 1% Penicillin/Streptomycin (Invitrogen, United States). After 3 days, non-adherent cells were removed and media was changed to osteogenic media containing 100 μM β-glycerophosphate, 2.84 μM ascorbic acid, and 10 nM dexamethasone. After 1 week in osteogenic media, stromal cultures were either lysed with Trizol (Invitrogen, United States) for RNA or stained for alkaline phosphatase activity (Sigma).

In osteoblast function experiments, ST2D cells were treated with *Tle4*-specific (T4KD) or scramble control shRNA (SCR) *via* lentiviral expression. One week after spinoculation, GFP + ST2D cells were selected using FACS Aria (BD, United States) and cultured in 6-well plates. Upon reaching confluence, ST2D cells were cultured in osteogenic media, with or without 350 ng/mL doxycycline. After 2 days, ST2D cultures were lysed with Trizol (Invitrogen, United States) for RNA. In a separate experiment RNA was harvested from ST2D cells cultured after stimulation with 350 ng/mL doxycycline at 24, 48, and 72 h.

### Expression Analysis *via* qRT-PCR

RNA was harvested from whole bone lysate, stromal cultures, or ST2D cell culture using Trizol (Invitrogen, United States). Expression levels of select differentially expressed genes and others of interest were performed *via* qRT-PCR as previously described (Wheat et al., 2014). Briefly, the RNA was reversed transcribed using the M-MLV Reverse transcriptase kit (Invitrogen, United States), followed by the quantitative analysis using either SYBR Green system (Bio-Rad, United States) or predesigned TaqMan Gene Expression Assay (Applied Biosystems, United States). Primer sequences for SYBR Green assays are listed in Supplementary Table 1. Tle4 expression was assayed using the TaqMan Gene Expression Assay (Tle4: Mm01196934). The expression levels of genes of interest were normalized to the expression levels of the 18S housekeeping gene.

### Mineral Apposition Rate Assay

For mineral apposition assay, 2-month old T4F and T4F cre littermates were irradiated with two doses of 450cGy and subsequently transplanted with 1 × 10^4^ Lineage- c-Kit + Sca-1 + (LKS) cells from 2-month old wild-type C57BL/6 mice *via* tail vein injection. These transplanted wild-type LKS cells were isolated using a FACS Aria (BD, United States). Eight weeks after transplant, *Tle4* excision was induced by three pIpC (Sigma) intraperitoneal injections at a dose of 15 mg/kg every 48 h. Four weeks after *Tle4* excision, recipient mice were pulsed with 20 mg/kg calcein (Sigma- Aldrich, United States) *via* intraperitoneal injection. After 1 week, recipients were given 30 mg/kg demeclocycline (Sigma- Aldrich, United States) *via* intraperitoneal injection. Femurs were harvested 3 days after demeclocycline injection and fixed in 70% ethanol. Femurs were processed for resin embedding for mineral apposition rate analysis and for immunohistochemistry staining of Runx2 (PA5-86506, ThermoFisher Scientific, Waltham, MA, United States), Oc (MBS2003553, MyBiosource, San Diego, CA, United States) and β-catenin (ab6302, Abcam, Cambridge, United Kingdom) (Bouxsein et al., 2010).

### MicroCT Bone Analysis

Microcomputed tomographic analysis (microCT) was performed on a subset of lumbar vertebrae. The femora were scanned at a resolution of 6 μm using a Scanco-35 microCT (Scanco United States, Inc., Southeastern, PA, United States). Each scan included a phantom containing air, saline and a bone reference material (1.18 g/cm3) for conversion of Houndsfield units to mineral density in g/cm3. Reconstruction of the individual projections to computed tomography volume data was performed using instrument software. Specimen-specific thresholds were determined by first selecting a volume of interest, generating the attenuation histogram, and determining the threshold that segments mineralized tissue from background. Properties determined included medullary bone mineral density (BMD), cortical bone thickness, AP distance, vertebral pedicle length, and trabecular bone volume fraction (trabecular bone volume to total volume ratio, BV/TV) (Bouxsein et al., 2010).

### Statistics

Analyses used student’s unpaired *t*-test with Graphpad Prism for comparing two genotypes (Graph-pad Software, La Jolla, CA, United States). Data are presented as floating bars showing the minimum to maximum values or scatter plots, and values of *p* < 0.05 are considered statistically significant.

## Results

### Loss of *Tle4* Leads to Defective Bone Development

**FIGURE 1:**
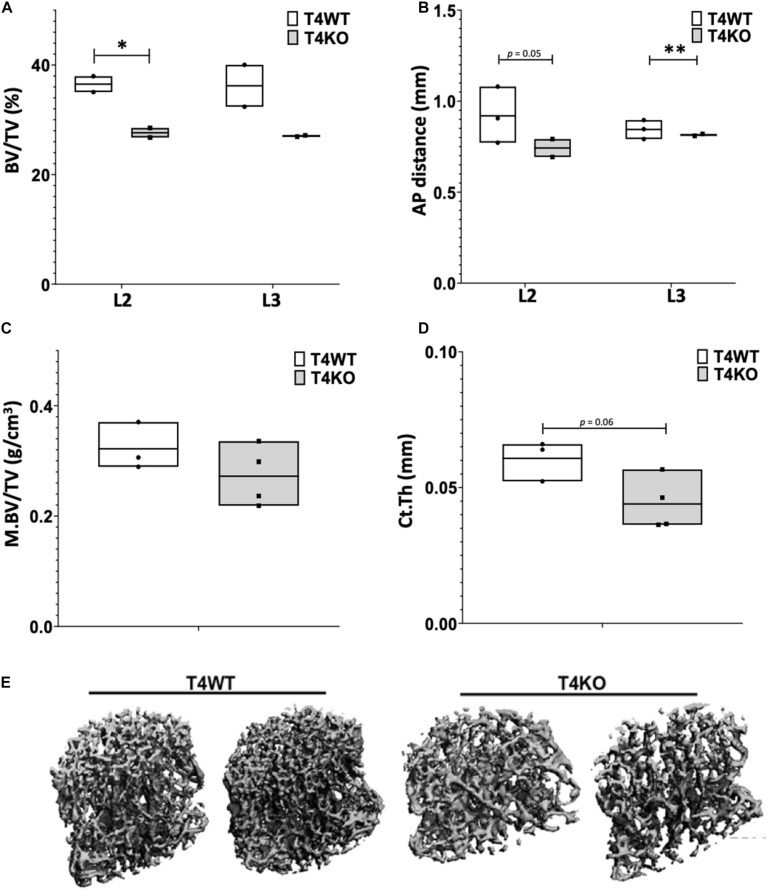
T4KO mice exhibit deficiencies in bone formation. MicroCT measurements of **(A)** BV/TV and **(B)** AP distance in L2 and L3 vertebrae of T4KO and T4WT littermates demonstrate decreased bone density and spinal canal diameters (*n* = 2 biologic duplicates per arm; ^∗^*p* < 0.05, ^∗∗^*p* < 0.01, Student *t*-test). Femurs collected from 9-day old T4KO and T4WT littermates were analyzed for **(C)** medullary bone density and **(D)** cortical thickness. Both measurements show decreased trend in T4KO vs. T4WT mice (*n* = 3–4 mice per arm, technical duplicates). **(E)** MicroCT image reconstructions of trabecular bone in L3 vertebrae of 3-to-4 weeks old T4WT and T4KO mice, illustrating decreased bone density and trabecular bone formation in *Tle4* null mice (*n* = 2 biologic duplicates per arm).

To assess bone formation in *Tle4* null mice, we performed microCT analysis of lumbar vertebrae of 3-day old T4KO and wild-type (T4WT) littermates. T4KO mice exhibit decreased trabecular bone volume fraction (BV/TV) and AP distance measurements compared to T4WT counterparts, suggesting loss of *Tle4* is associated with decreased bone density and vertebral pedicle length (Figures 1A,B). Additionally, femurs of 8 to 9-day-old T4KO and T4WT littermates were harvested for microCT analysis of medullary and cortical bone (Figures 1C,D). This analysis showed T4KO mice have reduced medullary bone density (M.BV/TV) and cortical bone thickness (Ct.Th). Moreover, microCT image reconstructions of trabecular bone in L3 vertebrae of 3 to 4-week old T4KO and T4WT littermates further illustrate decreased bone calcification and increased trabecular space in T4KO vertebrae compared to those of T4WT (Figure 1E). This is consistent with previous findings that loss of *Tle4* is associated with deficits in vertebral and long bone formation.

### Dynamic Bone Formation and Osteoblast Deficiencies in Conditional *Tle4* Knockout Mouse

**FIGURE 2:**
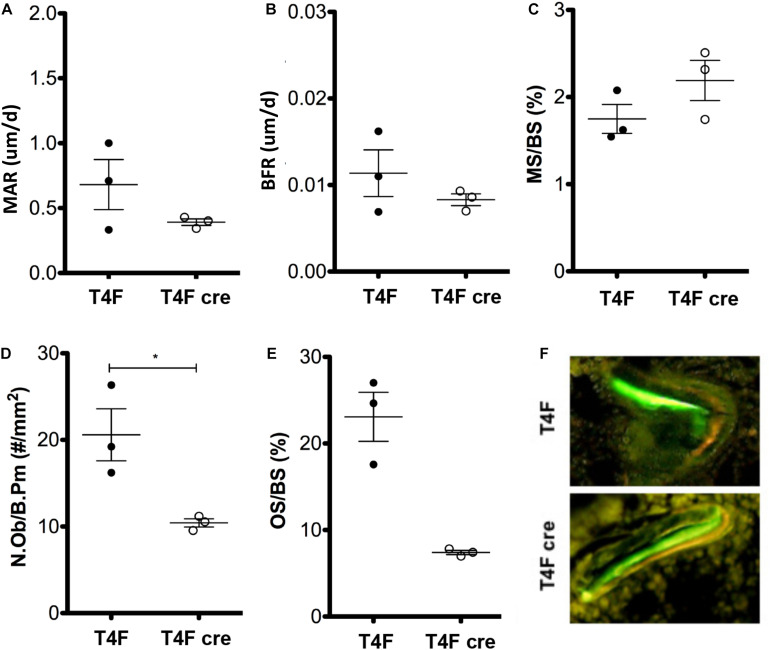
MAR assay and trichrome staining results indicate contrasting effects of *Tle4* loss on bone formation and calcification. T4F cre mice of 8-to-9 days old have decreased **(A)** MAR and **(B)** BFR rates, measurements of average bone calcification per osteoblast and total bone formation, respectively, while having similar **(C)** ratio of mineralizing to bone surface ratios. **(D)** T4F cre mice also had less N.Ob/B.Pm values, an estimate of the number of osteoblasts per area of bone surface. **(E)** Trichrome staining reveals T4F cre mice have significantly reduced osteoid compared to T4F controls (*n* = 3 mice per arm; *p < 0.05, Student *t*-test). **(F)** Representative image demonstrating decreased bone formation in T4F cre mice vs. T4F mice *via* MAR double-stain of calcein and demeclocycline.

To further characterize the effects of *Tle4* loss on bone development, we pursued a dynamic double-label mineral apposition rate (Calvi et al., 2003) assay using mice that have *loxP* target sites flanking exon 2 of *Tle4* with or without Cre recombinase driven by the *Mx1* promoter (T4F cre and T4F, respectively). In order to isolate the effects of *Tle4* loss to bone marrow mesenchymal cells we replaced the bone marrow hematopoietic cells with wild type cells by bone marrow transplantation prior to knockout of Tle4 by pIpC in these Mx1-Cre expressing mice. The high degree of knockout efficiency of exon 2 of Tle4 in these conditional T4F-cre mice was demonstrated in harvested bone marrow of similarly pIpC treated T4F-cre compared to T4F mice lacking Mx1-cre (Supplementary Figure 1). The MAR assay revealed T4F cre mice have multiple lower dynamic parameters of bone formation (Figures 2A–F). While ratios of mineralizing to bone surface areas (MS/BS) were similar, MAR and bone formation rates (BFR) were lower in T4F cre mice compared to their control T4F counterparts (Figures 2A–C). Additionally, T4F cre mice had lower numbers of osteoblasts per given bone perimeter area (Figure 2D). Combined with lower osteoid to bone surface area ratios (Figure 2E), these results point toward an association between decreased bone formation and conditional loss of *Tle4* in adult mice.

### Bone Defects Due to Tle4 Loss May Be Mediated Through Dysregulation of Canonical Regulators of Bone Development

**FIGURE 3:**
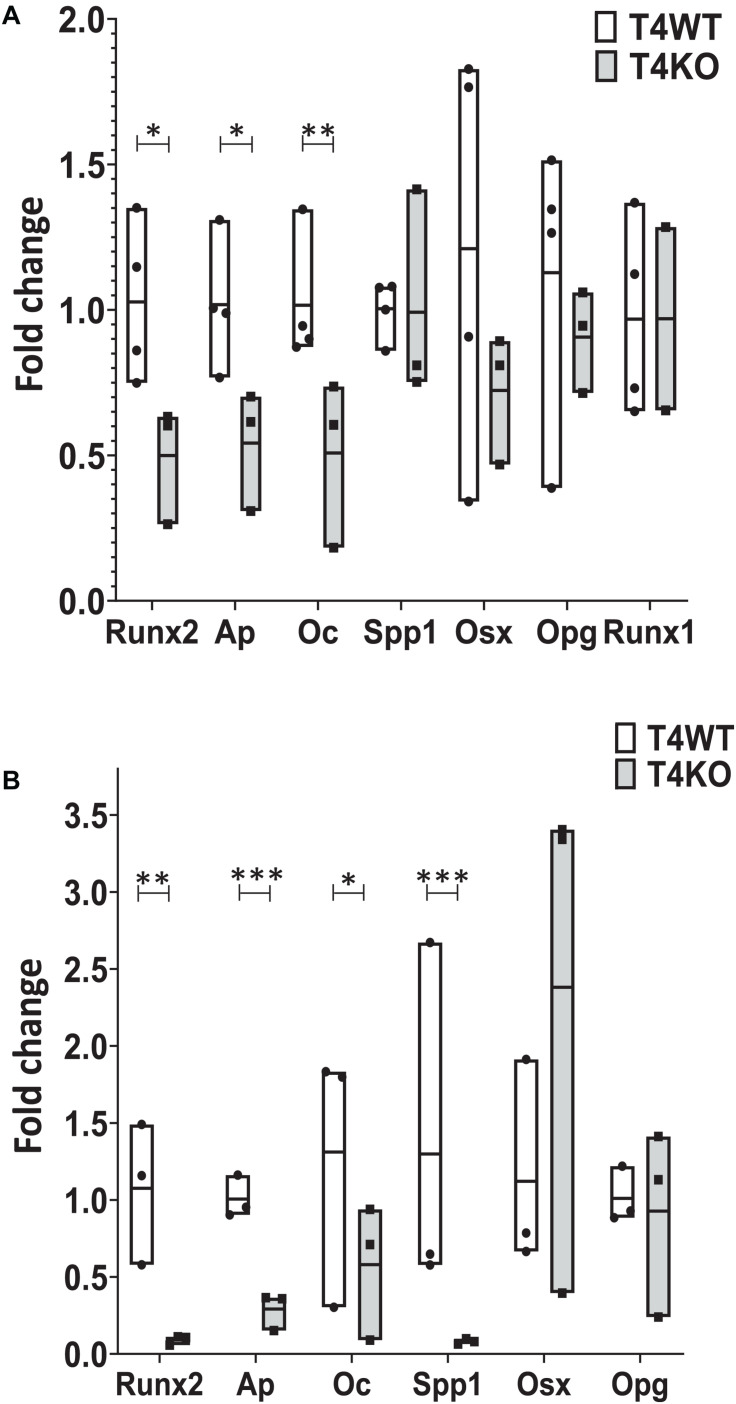
Loss of *Tle4* affects expression of osteoblast function and differentiation regulators. **(A)** qRT-PCR using RNA from 1-week old T4WT and T4KO flushed whole bone lysate shows T4KO has decreased expression of *Ap, Runx2, and Oc* (*n* = 6 biologic replicates, technical triplicates; *p < 0.05, **p < 0.01, Student *t*-test). **(B)** Stromal cultures from T4WT and T4KO were lysed for qRT-PCR analysis, which shows significantly reduced levels of *Runx2*, *Ap*, *Oc*, and *Spp1* expression in T4KO compared to T4WT (*n* = 3 biologic replicates per arm, technical triplicates; *p < 0.05, **p < 0.01, ***p < 0.001, and Student *t*-test).

Given the defective bone and bone marrow phenotype seen in T4KO mice, we hypothesized that loss of *Tle4* may affect osteoblast function and development. To assess this, we first harvested RNA from flushed whole bone lysates of 1-week-old T4KO and T4WT littermates. Expression analysis, using qPCR, revealed T4KO bone had significantly lower levels of *Ap*, *Runx2*, and *Oc* expression (Figure 3A). *Ap* is often used as a proxy for osteoblast function while *Runx2* and *Oc* have previously been connected to osteoblast maturation and bone anabolic regulation (Jang et al., 2012). To minimize bone cell heterogeneity, crushed 1-week-old T4KO and T4WT femurs were cultured in osteogenic media to generate osteoblast stromal cultures. T4KO stromal cultures demonstrated significant decreases in osteoblast genes, including *Runx2*, *Ap*, *Oc*, and *Spp1* (Figure 3B). Moreover, alkaline phosphatase staining qualitatively showed a trend toward decreased alkaline phosphatase activity in T4KO stromal cells (Supplementary Figure 2).

Our T4KO results revealed that absence of *Tle4* was associated with significant reductions of *Runx2*, suggesting T4KO-associated bone abnormalities might be due to decreased *Runx2* expression. To further elucidate the time course of Tle4-mediated effects on bone development factors through the Runx axis and considering the recent data on the Runx1 involvement in bone development (Tang et al., 2020), we assessed the expression levels of Runx1 in the 1-week-old T4KO and T4WT littermates, and found that the absence of Tle4 does not affect the expression levels of *Runx1* (Figure 3A) suggesting the calcification defects in Tle4 KO mice are more likely explained by Runx2 inhibition.

**FIGURE 4:**
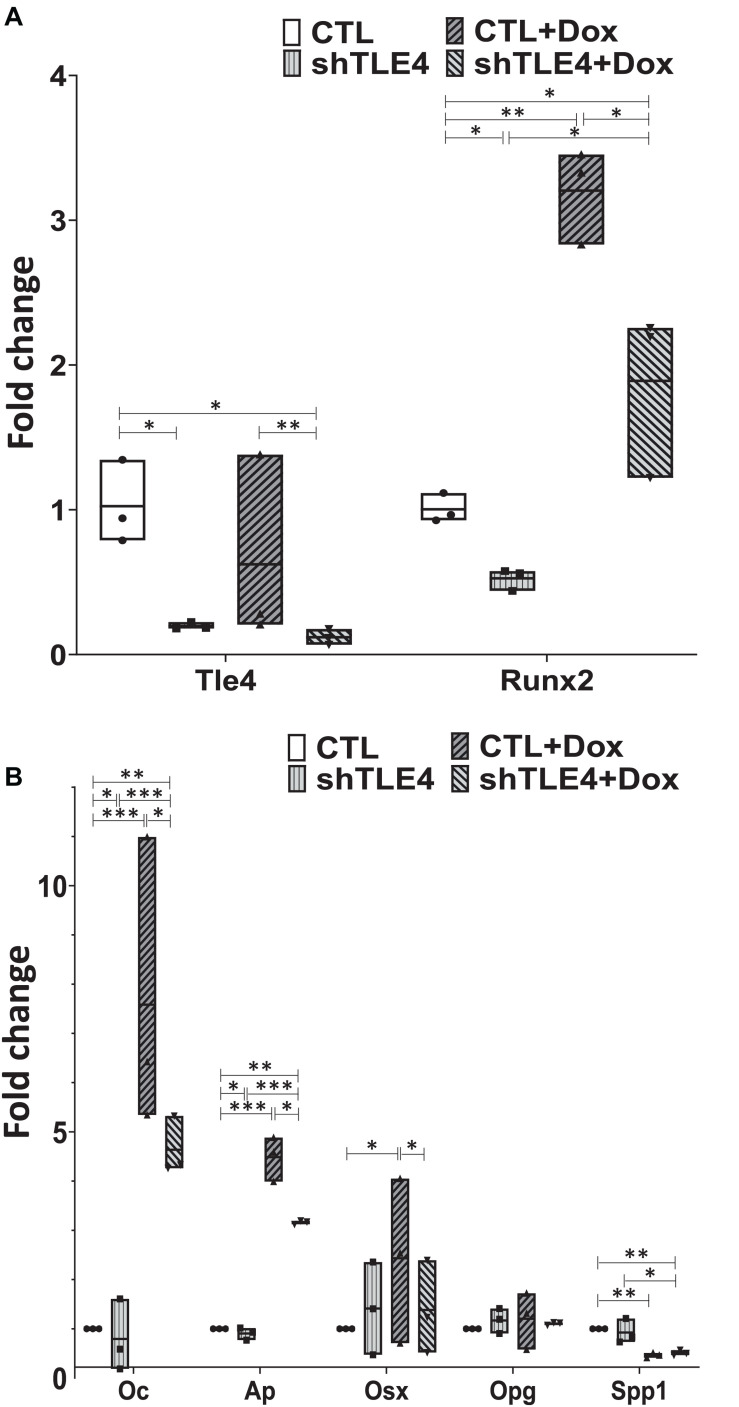
*Tle4* knockdown in ST2D mouse mesenchymal cells demonstrates similarities in aberrant expression of osteoblast regulator genes seen in osteoblast cultures derived from T4KO mice. **(A)** qRT-PCR using RNA harvested from *Tle4* knockdown (shTle4) and control (CTL) ST2D cells using scrambled shRNA confirm effective *Tle4* knockdown and *Runx2* induction after 48 hr of doxycycline induction which remains decreased compared to the scramble controls. **(B)** qRT-PCR experiments show expression of *Ap* and *Oc* are significantly reduced in *Tle4* knockdown ST2D cells compared to control. Doxycycline-induced expression of *Runx2* significantly increases expression of these genes compared to DMSO control, remaining however, in lower levels compared to scramble (*n* = 3 biologic replicates per arm, technical triplicates; *p < 0.05, **p < 0.01, ***p < 0.001, and Student *t*-test).

**FIGURE 5:**
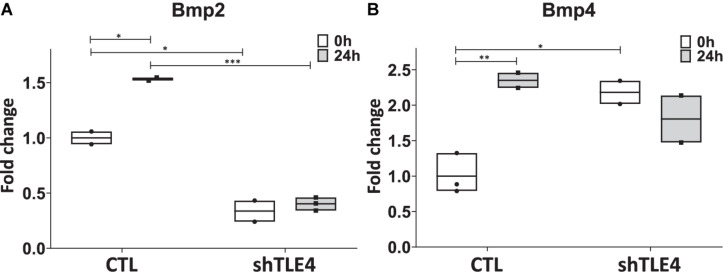
*Tle4* knockdown in ST2D cells cause aberrant levels of *Bmp2* and *Bmp4* expression in response to *Runx2* expression. **(A)** Twenty-four hours after *Runx2* induction, upregulation of Bmp2 in control (CTL) cells. Knockdown of *Tle4* represses Bmp2 expression and blocks upregulation with Runx2 induction. **(B)** Induction of *Runx2* also leads to an upregulation of *Bmp4* in control cells. This upregulation is not seen in the presence of *Tle4* knockdown (*n* = 3 biologic replicates per arm, technical triplicates; *p < 0.05, **p < 0.01, ***p < 0.001, and Student *t*-test).

To better understand the mechanisms underlying Tle4 effect on bone development and the interplay with Runx2, we turn to an *in vitro* system using mouse mesenchymal ST2 cells stably transformed with a doxycycline-inducible *Runx2* vector (ST2D) (Dayyani et al., 2008; Baniwal et al., 2012). In these ST2D cells the addition of doxycycline leads to an induction of *Runx2* expression (Figure 4A). In this system we evaluated the effects of knocking down Tle4 expression *via* lentiviral delivery of *Tle4*-specific shRNA. Expression analysis using qPCR demonstrated over 80% reduction in *Tle4* message *via* shRNA in both doxycycline and DMSO cultures and showed that ectopic expression of *Runx2* in the absence of Tle4 shRNA did not significantly affect *Tle4* expression levels (Figure 4A). In this system we demonstrated *Tle4* knockdown reduced endogenous *Runx2* expression levels by approximately 50% in ST2D cells cultured in control DMSO media. This result correlated nicely with that found *in vivo*. The addition of doxycycline significantly increased *Runx2* expression levels. *Tle4* knockdown *via* shRNA was not able to prevent this increase driven from a lentiviral promoter, though the increase was blunted, likely reflecting repression of *Runx2* from the endogenous promoter. Induction of *Runx2* by doxycycline resulted in a significant increase in the expression of *Oc, Ap*, and *Osx* after 48 h, and this increase was significantly decreased in *Tle4* knockdown arms (Figure 4B). To further understand the relationship between *Tle4* and *Runx2*, we queried expression levels of *Bmp2* and *Bmp4*, given their previously described roles as regulators of *Runx2* and normal skeletal development (Bandyopadhyay et al., 2006; Krishnan et al., 2006). We demonstrated that loss of *Tle4* caused a significant reduction of *Bmp2* expression at baseline and prevented upregulation in response to Runx2 induction. Interestingly, levels of *Bmp4* were increased with *Tle4* knockdown, but the upregulation seen with Runx2 expression was blocked (Figure 5).

**FIGURE 6:**
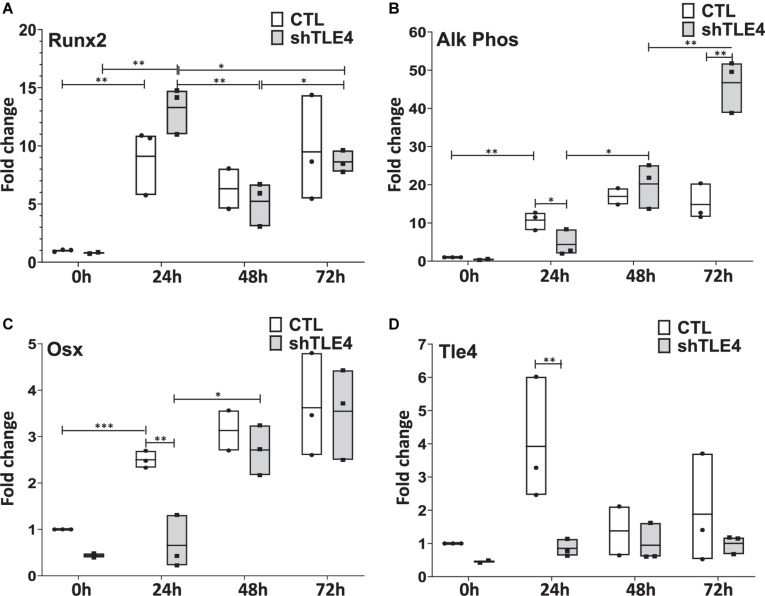
Time-course gene expression study demonstrates altered expression of *Runx2* and its bone-related targets due to *Tle4* knockdown (shTLE4) compared with control cells (CTL) in ST2D cells at 0, 24, 48, and 72 h. **(A)** ST2D cells that contain a doxycycline inducible Runx2 gene demonstrated maximal Runx2 induction after 24 h. **(B)** This is associated with an upregulation of alkaline phosphatase that was slightly delayed with Tle4 knockdown. **(C)** Tle4 knockdown also slows the induction of the osteoblast-specific transcription factor Osterix (Osx). **(D)** The knockdown of Tle4 achieved with a Tle4 specific shRNA was demonstrated at the different time points in this experiment. (0—knockdown to 37%, 24 h to 22%, 48 h to 69%, 72 h to 53%) (*n* = 3 biologic replicates per arm, technical triplicates; *p < 0.05, **p < 0.01, ***p < 0.001, and Student *t*-test).

Extended time-course experiments demonstrate that *Tle4* knockdown creates an initial surge of Runx2 expression by 24 h of knockdown compared to control, followed by decreased *Runx2* levels at 48 and 72 h; consistent with the above observations (Figure 6). However, we found decreased expression levels of *Runx2*-mediated regulators of bone, including *Alp* and *Osx* at 24 h. This suggests the loss of Tle4 blunted the ability of Runx2 to upregulate Osx and Alp expression at early time points. By 72 h after *Tle4* knockdown, the differential expression of these genes is lost, suggesting a time-delay of *Alp* and *Osx* induction due to *Tle4* knockdown. These experiments support a role of Tle4 in regulating Runx2 and, subsequently, its target genes canonically associated with osteoblast differentiation and function.

**FIGURE 7:**
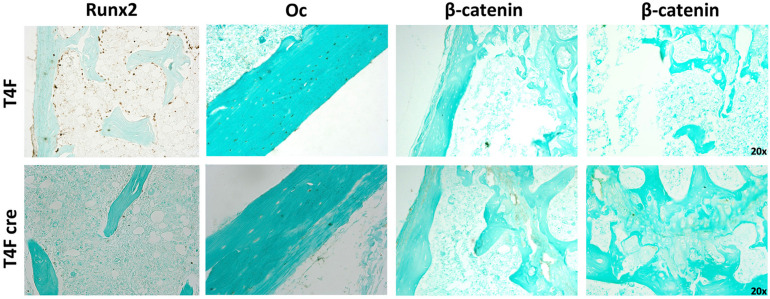
T4F cre mice have significantly reduced levels of key regulators of bone development and osteoblast function. Immunohistochemistry assay using femurs harvested from T4F cre mice of 8-to-9 days old in MAR assay reveals decrease in Runx2 and Oc levels and increased β-catenin signals in the epiphyseal and cortical areas compared to T4F control counterparts (*n* = 3 mice per arm, representative images).

Immunohistochemistry using femurs from mice used in MAR assay revealed decreased amounts of Runx2 and Osteocalcin in T4F cre mice compared to control (Figure 7). In T4F mice, Runx2 positivity can be seen in numerous osteoid-lining cells, suggestive of Runx2-positive periosteal osteoblasts. The frequency and localization of these Runx2-positive cells dramatically drops in T4F cre mice. Similarly, osteocalcin positivity in cortical bone seen in T4F mice is not evident in the conditional *Tle4* knockout mice. In addition to *Runx2*, β-catenin-mediated Wnt signaling has also been previously described as an important mediator of bone development and osteoblast differentiation (Hill et al., 2005; Cohen, 2006; Rodda and McMahon, 2006; Kook et al., 2015). Given previous reports implicating *Tle4* as a negative regulator of Wnt signaling, we queried β-catenin levels *via* immunohistochemistry in bones of the T4F and T4F cre mice used in the above MAR assay as an exploratory objective. T4F cre femurs showed increased β-catenin signals in the epiphyseal and cortical areas. While T4F cre and T4KO mice display bone calcification and osteoblast function defects, loss of *Tle4* in bone leads to increased β-catenin levels, which is consistent with previous reports implicating *Tle4* as a repressor of Wnt signaling. However, using the above-described ST2D system, we were not able to find a *Tle4* knockdown-dependent change in expression of canonical *Wnt* target gene expression (data not shown).

## Discussion

The Groucho/TLE family of proteins has been extensively studied in Drosophila where it has been termed a master regulatory gene in development *via* its interaction with a number of important signaling pathways including Notch and Wnt and also can be recruited by transcription factors members of Hex, Runx, Nkx, Lef1/Tcf, Pax, Six and c-myc (Jennings and Ish-Horowicz, 2008; Agarwal et al., 2015). Our understanding of the roles of this protein family in vertebrate development is limited. The novel *Tle4* null mouse provides valuable insight into the previously unappreciated roles of *Tle4* in mammalian vertebrates regarding bone maturation, medullary hematopoiesis and HSPC maintenance. One of the striking abnormalities in the T4KO mice is a decreased calcification of the skeleton (Wheat et al., 2014). This impaired ossification is apparent in both membranous and endochondral bones by birth. The above phenotypes are more intense and progressive in an age-dependent manner in T4KO mice than in *Grg5* null mice lacking a truncated member of the Groucho/TLE family (Wheat et al., 2014). Our earlier characterization of Tle4 KO mice suggested *Tle4* also affects osteoclast function as demonstrated by an increase in osteoclasts by tartrate-resistance acid phosphatase (TRAP) staining (Wheat et al., 2014). In the current study microCT and MAR assay measurements indicate loss of Tle4 impaired bone formation, calcification, and osteoid production. The lethality of *Tle4* null mice and the effects in hematopoietic cells and observed degradation of the bone marrow niche made it technically challenging to isolate the effect on osteoblasts in germline knockout mice. Conditional *Tle4* mice transplanted with normal bone marrow hematopoietic cells served as a proxy for osteoblast-specific effects of *Tle4* loss. Mx1-cre system has been demonstrated as one of the most commonly “deleter strain” in experimental hematology (Velasco-Hernandez et al., 2016). Previous work by Park et al. (2012) have demonstrated the contributory role of *Mx1*-expressing bone mesenchymal cells toward the generation of new osteoblasts responsible for new bone formation, supporting the use of the *Mx1*-Cre model in our experiments to examine the role of *Tle4* loss in osteoblast function (Park et al., 2012). The concordance of observations seen in these models and studies strongly suggest that *Tle4* may affect osteoblasts and other periosteal cells that are responsible for bone production and maintenance.

In the context of our previous work identifying hematopoietic defects in *Tle4* null mice, we have attempted to determine whether the bone abnormalities due to *Tle4* loss can be attributable to dysfunctional osteoblasts. We had shown Tle4 loss significantly impairs LSK differentiation into granulocyte, monocyte, macrophage progenitors and LSK self-renewal and adversely affects the integrity of bone marrow niche and stroma (Wheat et al., 2014). Osteoblasts are known to play a critical role in maintaining the bone marrow niche (Calvi et al., 2003; Asada et al., 2013; Fulzele et al., 2013; Even et al., 2021). In this current work qRT-PCR analysis using T4KO mouse samples from flushed whole bones and cultured stromal cells revealed significantly decreased expression of various transcription factors and regulators responsible for osteoblast function and differentiation, including *Ap* and *Oc*—both frequently used proxies for describing osteoblast function. Interestingly, osteocalcin is one of the main components of ground substance that, together with Type 1 collagen, constitute the bone matrix (Hill et al., 2005; Asada et al., 2013; Fulzele et al., 2013). *Osx* demonstrates a multifunctional role on osteoblast differentiation, growth and homeostasis, since its deletion in several time points postnatally in growing and adult bones causes defects in maturation, morphology and function of osteocytes (Zhou et al., 2010; Liu et al., 2020). Decreased *Oc* expression may lend insight into a physiologic basis for the *Tle4* knockout-induced bone phenotype.

Wnt signaling has been described as a central mediator of bone formation (Hill et al., 2005; Cohen, 2006; Rodda and McMahon, 2006; Kook et al., 2015). Surprisingly, we observed defective bone formation and calcification in T4KO mice, in which Wnt signaling is expected to be activated especially given higher levels of β-catenin signal as determined by immunohistochemistry (Kronenberg, 2003; Chodaparambil et al., 2014). However, previous studies have shown that there is an intimate relationship between the timing of Wnt signaling and normal osteoblast differentiation; and thus, constitutive or increased Wnt signaling at an inappropriate stage of osteoblast development may be detrimental to normal bone growth (Rodda and McMahon, 2006; Janeway and Walkley, 2010). Alternatively, the detrimental effects of *Tle4* loss on Runx2 activity might outweigh the effects of Wnt activation on osteoblast differentiation and calcification. Our results do not preclude the possibility that loss of *Tle4* is responsible for the bone defects through other mechanisms and the potential effects of paracrine hormonal or *Tle4* levels in other non-*Mx1*-expressing compartments.

The ST2D system provided insight into potential molecular mechanisms that may explain our findings, including not only Wnt signaling but also dysregulation of Bmp signaling and consequently *Runx2* expression. Previous studies have demonstrated loss of function of *Bmp2* and *Bmp4* impair bone condensation and skeletal development (Bandyopadhyay et al., 2006; Krishnan et al., 2006; Wu et al., 2016). There is an interplay between BMP and RUNX2 in regulating osteoblast differentiation (Lowery and Rosen, 2018). BMP signaling is required for transcriptional activity of Runx2 and Runx2 enhances the sensitivity of cells to BMPs (Phimphilai et al., 2006).

Our experiments indicate there is a decrease in *Runx2* expression in T4KO bone, T4KO stromal cells, and in ST2D cells with *Tle4* knockdown in a time dependent manner. Previous studies have shown that TLE proteins are capable of interacting with Runx2, a critical regulator of bone development and maturation (McLarren et al., 2000; Choi et al., 2001; Kaul et al., 2015). The RUNX protein family is known to form co-repressor complexes with TLE proteins (Javed et al., 2000). Thus, the TLE proteins might affect both *Runx2* expression as well as the activity of the Runx2 protein. The TLE effect on *Runx2* expression could reflect interference of Runx2 transcriptional autoregulation (Drissi et al., 2000). In our studies, even if the experiments do not distinguish whether the blunting in the expression of these osteoblast genes with *Tle4* knockdown is due to decreased *Runx2* expression or decreased function of the Runx2 protein in the absence of Tle4, the differential expression levels of *Runx2*-mediated regulators of bone development are most likely a downstream effect consequent of the decreased endogenous *Runx2* expression after *Tle4* knockdown. *Runx2* null mice demonstrate bone phenotypes similar, but more severe than our T4KO mouse: expiring at birth and completely missing skeletal and bone development, owing to defective osteoblast maturation (Komori et al., 1997; Otto et al., 1997; Okura et al., 2014). Previous studies have shown the importance of *Runx2* in normal bone development, as *Runx2* null mice lack bone ossification and osteoblast differentiation (Cohen, 2006). The similarity of *Tle4* null mice to *Runx2* null mice suggested the loss of *Tle4* might either impair the function or the expression of *Runx2*. The less severe effect observed with *Tle4* knockout could reflect complementary effects from the expression of other *Tle* family members along with Runx2 residual expression which thus induce the osteoblastic genes expression later during bone development in the Tle4 loss background, in contrast to the early in time severe effects of Tle4 loss in bone development. Additional studies are required to further characterize the interaction and potential regulatory role of *Tle4* on *Runx2* expression levels and function as it may relate to the defective bone development in the absence of Tle4. While direct functional interactions between RUNX and TLE have been described (Westendorf, 2006) and a possibility of the requirement of such a direct active interaction in the early osteoblastic development is suggested in our time dependent experiments, additional experiments may reveal further insight into whether TLE exerts a direct regulatory effect on RUNX transcription, stability or targets.

## Data Availability Statement

The original contributions presented in the study are included in the article/Supplementary Material, further inquiries can be directed to the corresponding author/s.

## Ethics Statement

The animal study was reviewed and approved by Guide for the Care and Use of Laboratory Animals of the National Institutes of Health and approved by the Massachusetts General Hospital Institutional Animal Care and Use Committee.

## Author Contributions

DS, TS, and ET: study conception and design, writing manuscript, and study supervision. TS, ET, CH, RY, CR, and PG: study conduct and data acquisition. TS, ET, CR, PG, and DS: methodology development and data analysis and interpretation. ET and DS: revising manuscript. All authors contributed to the article and approved the submitted version.

## Conflict of Interest

The authors declare that the research was conducted in the absence of any commercial or financial relationships that could be construed as a potential conflict of interest.

## Publisher’s Note

All claims expressed in this article are solely those of the authors and do not necessarily represent those of their affiliated organizations, or those of the publisher, the editors and the reviewers. Any product that may be evaluated in this article, or claim that may be made by its manufacturer, is not guaranteed or endorsed by the publisher.

## References

[B1] Agarwal M. Kumar P. Mathew S. J. (2015). The Grpucho/Transducin-like enhacer of split protein family in animal development. *IUBMB Life* 71: 1824. 10.1002/iub.1395 PMC7015701 26172616

[B2] Aguila H. Rowe D. (2005). Skeletal development, bone remodeling, and hematopoiesis. *Immunol. Rev.* 208 7–18. 10.1111/j.0105-2896.2005.00333.x 16313337

[B3] Asada N. Katayama Y. (2014). Regulation of helatopoiesis in endosteal microenvironments. *Int. J. Hematol.* 99 679–684. 10.1007/s12185-014-1583-1 24760425

[B4] Asada N. Katayama Y. Sato M. Minagawa K. Wakahashi K. Kawano H. (2013). Matrix-embedded osteocytes regulate mobilization of hematopoietic stem/progenitor cells. *Cell Stem Cell* 12 737–747. 10.1016/j.stem.2013.05.001 23746979

[B5] Bandyopadhyay A. Tsuji K. Cox K. Harfe B. D. Rosen V. Tabin C. J. (2006). Genetic analysis of the roles of BMP2, BMP4, and BMP7 in limb patterning and skeletogenesis. *PLoS Genet.* 2:e216. 10.1371/journal.pgen.0020216 17194222 PMC1713256

[B6] Baniwal S. K. Shah P. K. Shi Y. Haduong J. H. Declerck Y. A. Gabet Y. (2012). Runx2 promotes both osteoblastogenesis and novel osteoclastogenic signals in ST2 mesenchymal progenitor cells. *Osteoporos. Int.* 23 1399–1413. 10.1007/s00198-011-1728-5 21881969 PMC5771409

[B7] Bianco P. (2011). Bone and hematopoietic niche: atale of two stem cells. *Blood* 117 5281–5288. 10.1182/blood-2011-01-315069 21406722

[B8] Bouxsein M. L. Boyd S. K. Christiansen B. A. Guldberg R. E. Jepsen K. J. Muüller R. (2010). Guidelines for assessment of bone microstructure in rodents using micro-computed tomography. *J. Bone Miner. Res.* 25 1468–1486. 10.1002/jbmr.141 20533309

[B9] Calvi L. Adams G. Weibrecht K. Weber J. M. Olson D. P. Knight M. C. (2003). Osteoblastic cells regulate the haematopoietic stem cell niche. *Nature* 425 841–845. 10.1038/nature02040 14574413

[B10] Chen Q. Courey A. (2000). Groucho/TLE family proteins and transcriptional repression. *Genes Chromosomes Cancer* 249 1–16. 10.1016/s0378-1119(00)00161-x 10831834

[B11] Chodaparambil J. V. Pate K. T. Hepler M. R. Tsai B. P. Muthurajan U. M. Luger K. (2014). Molecular functions of the TLE tetramerization domain in Wnt target gene repression. *EMBO J.* 33 719–731. 10.1002/embj.201387188 24596249 PMC4000089

[B12] Choi J. Y. Pratap J. Javed A. Zaidi S. K. Xing L. Balint E. (2001). Subnuclear targeting of Runx/Cbfa/AML factors is essential for tissue-specific differentiation during embryonic development. *Proc. Natl. Acad. Sci. U.S.A.* 98 8650–8655. 10.1073/pnas.151236498 11438701 PMC37490

[B13] Cohen M. M. Jr. (2006). The new bone biology: pathologic, molecular, and clinical correlates. *Am. J. Med. Genet. A* 140 2646–2706. 10.1002/ajmg.a.31368 17103447

[B14] Dayyani F. Wang J. Yeh J. R. Ahn E. Y. Tobey E. Zhang D. E. (2008). Loss of TLE1 and TLE4 from the del(9q) commonly deleted region in AML cooperates with AML1-ETO to affect myeloid cell proliferation and survival. *Blood* 111 4338–4347. 10.1182/blood-2007-07-103291 18258796 PMC2288729

[B15] Despars G. St-Pierre Y. (2011). Bidirectional interactions between bone metabolism and hematopoiesis. *Exp. Hematol.* 39 809–816. 10.1016/j.exphem.2011.04.008 21609752

[B16] Drissi H. Luc Q. Shakoori R. Chuva De Sousa Lopes S. Choi J. Y. Terry A. (2000). Transcriptional autoregulation of the bone related CBFA1/RUNX2 gene. *J. Cell. Physiol.* 184 341–350. 10.1002/1097-4652(200009)184:3<341::aid-jcp8>3.0.co;2-z 10911365

[B17] Even J. Yi L. Chang C. K. Rossi F. M. V. (2021). The parathyroid hormone-dependent activation of osteoblasts enhances hematopoietic stem cell migration and reduces their engraftment abilities. *bioRxiv* [Preprint]. 10.1101/2021.03.04.433901

[B18] Fulzele K. Krause D. S. Panaroni C. Saini V. Barry K. J. Liu X. (2013). Myelopoiesis is regulated by osteocytes through Gsalpha-dependent signaling. *Blood* 121 930–939. 10.1182/blood-2012-06-437160 23160461 PMC3567340

[B19] Galan-Diez M. Kousteni S. (2018). A bone marrow niche-derived molecular switch between oestogenesis and hematopoiesis. *Genes Dev.* 32 324–326. 10.1101/gad.314013.118 29593065 PMC5900706

[B20] Garcia-Garcia A. de Castillejo C. L. Mendez-Ferrer S. (2015). BMSCs and hematopoiesis. *Immunol. Lett.* 168 129–135. 10.1016/j.imlet.2015.06.020 26192443

[B21] Hill T. P. Später D. Taketo M. M. Birchmeier W. Hartmann C. (2005). Canonical Wnt/beta-catenin signaling prevents osteoblasts from differentiating into chondrocytes. *Dev. Cell* 8 727–738. 10.1016/j.devcel.2005.02.013 15866163

[B22] Houschyar K. S. Tapking C. Borelli M. R. Popp D. Duscher D. Maan Z. N. (2019). Wnt pathway in bone repair and regeneration-what do we know so far. *Front. Cell Dev. Biol.* 6:170. 10.3389/fcell.2018.00170 30666305 PMC6330281

[B23] Janeway K. A. Walkley C. R. (2010). Modeling human osteosarcoma in the mouse: from bedside to bench. *Bone* 47 859–865. 10.1016/j.bone.2010.07.028 20696288

[B24] Jang W. G. Kim E. J. Kim D. K. Ryoo H. M. Lee K. B. Kim S. H. (2012). BMP2 protein regulates osteocalcin expression via Runx2-mediated Atf6 gene transcription. *J. Biol. Chem.* 287 905–915. 10.1074/jbc.m111.253187 22102412 PMC3256879

[B25] Javed A. Guo B. Hiebert S. Choi J. Y. Green J. Zhao S. C. (2000). Groucho/TLE/R-esp proteins associate with the nuclear matrix and repress RUNX (CBFa/AML/PEBP2a) dependent activation of tissue-specific gene transcription. *J. Cell Sci.* 113 2221–2231. 10.1242/jcs.113.12.2221 10825294

[B26] Jennings B. H. Ish-Horowicz D. (2008). The Groucho/TLE/Grg family of transcriptional co-repressors. *Genome Biol.* 9:205. 10.1186/gb-2008-9-1-205 18254933 PMC2395242

[B27] Kaul H. Hall B. K. Newby C. Ventikos Y. (2015). Synergistic activity of polarised osteoblasts inside condensations cause their differentiation. *Sci. Rep.* 5:11838. 10.1038/srep11838 26146365 PMC4491713

[B28] Kode A. Manavalan J. S. Mosialou I. Bhagat G. Rathinam C. V. Luo N. (2014). Leukaemogenesis induced by an activating beta-catenin mutation in osteoblasts. *Nature* 506 240–244. 10.1038/nature12883 24429522 PMC4116754

[B29] Komori T. (2019). Regulation of proliferation, differentiation and functions of osteoblasts by Runx2. *Int. J. Mol. Sci.* 20:1694. 10.3390/ijms20071694 30987410 PMC6480215

[B30] Komori T. Yagi H. Nomura S. Yamaguchi A. Sasaki K. Deguchi K. (1997). Targeted disruption of Cbfa1 results in a complete lack of bone formationowing to maturational arrest od osteoblasts. *Cell* 89 755–764. 10.1016/s0092-8674(00)80258-5 9182763

[B31] Kook S. H. Heo J. S. Lee J. C. (2015). Crucial roles of canonical Runx2-dependent pathway on Wnt1-induced osteoblastic differentiation of human periodontal ligament fibroblasts. *Mol. Cell. Biochem.* 402 213–223. 10.1007/s11010-015-2329-y 25618247

[B32] Kozhemyakina E. Lassar A. Zelzer E. (2015). A pathway to bone: signaling molecules and transcription factors involved in chondrocyte development and maturation. *Development* 142 817–831. 10.1242/dev.105536 25715393 PMC4352987

[B33] Krishnan V. Bryant H. U. Macdougald O. A. (2006). Regulation of bone mass by Wnt signaling. *J. Clin. Invest.* 116 1202–1209. 10.1172/jci28551 16670761 PMC1451219

[B34] Kronenberg H. (2003). Developmental regulation of the growth plate. *Nature* 423 332–335. 10.1038/nature01657 12748651

[B35] Le P. M. Andreeff M. Battula V. L. (2018). Osteogenic niche in the regulation of normal hematopoiesis and leukemogenesis. *Haematologica* 103 1945–1955. 10.3324/haematol.2018.197004 30337364 PMC6269284

[B36] Levanon D. Goldstein R. Bernstein Y. Tang H. Goldenberg D. Stifani S. (1998). Transcriptional repression by AML1 and FEF-1 is mediated by the TLE/Groucho corepressors. *Proc. Natl. Acad. Sci. U.S.A.* 95 11590–11595. 10.1073/pnas.95.20.11590 9751710 PMC21685

[B37] Liu Q. Li M. Wang S. Xiao Z. Xiong Y. Wang G. (2020). Recent advances of osterix transcription factor in osteoblast differentiation and bone formation. *Front. Cell Dev. Biol.* 8:601224. 10.3389/fcell.2020.601224 33384998 PMC7769847

[B38] Lowery J. Rosen V. (2018). The BMP pathway and its inhibitor in the skeleton. *Physiol. Rev.* 98 2431–2452. 10.1152/physrev.00028.2017 30156494

[B39] Ma X. Liu Y. Liu Y. Alexandrov L. B. Edmonson M. N. Gawad C. (2018). Pan-cancer genome and transcriptome analyses of 1,699 paediatric leukaemias and solid tumours. *Nature* 555 371–376. 10.1038/nature25795 29489755 PMC5854542

[B40] McLarren K. Lo R. Grbavec D. Thirunavukkarasu K. Karsenty G. Stifani S. (2000). The mammalian basic helix loop helix protein HES-1 binds to and modulates the transactivating function of the Runt-related factor Cbfa1. *J. Biol. Chem.* 275 530–538. 10.1074/jbc.275.1.530 10617648

[B41] Mukherjee S. Raje N. Schoonmaker J. A. Liu J. C. Hideshima T. Wein M. N. (2008). Pharmacologic targeting of a stem/progenitor population *in vivo* is associated with enhanced bone regeneration in mice. *J. Clin. Invest.* 118 491–504. 18219387 10.1172/JCI33102 PMC2213372

[B42] Okura H. Sato S. Kishikawa S. Kaneto S. Nakashima T. Yoshiba N. (2014). Runx2-I isoform contributes to fetal bone formation even in the absence of specific N-terminal amino acids. *PLoS One* 9:e108294. 10.1371/journal.pone.0108294 25244033 PMC4171521

[B43] Otto F. Thornell A. Crompton T. Denzel A. Gilmour K. C. Rosewell I. R. (1997). Cbfa1, a candidate gene for cleidocranial dysplasia syndrome, is essential for osteoblast differentiation and bone development. *Cell Stem Cell* 89 765–771. 10.1016/s0092-8674(00)80259-7 9182764

[B44] Park D. Spencer J. A. Koh B. I. Kobayashi T. Fujisaki J. Clemens T. L. (2012). Endogenous bone marrow MSCs are dynamic, fate-restricted participants in bone maintenance and regeneration. *Cell Stem Cell* 10 259–272. 10.1016/j.stem.2012.02.003 22385654 PMC3652251

[B45] Phimphilai M. Zhao Z. Boules H. Roca H. Franceschi R. T. (2006). BMP signaling is required for RUNX2-dependent induction of the osteoblast phenotype. *J. Bone Miner. Res.* 21 637–646. 10.1359/jbmr.060109 16598384 PMC2435171

[B46] Rauch D. A. Hurchla M. A. Harding J. C. Deng H. Shea L. K. Eagleton M. C. (2010). The ARF tumor suppressor regulates bone remodeling and osteosarcoma development in mice. *PLoS One* 5:e15755. 10.1371/journal.pone.0015755 21209895 PMC3012707

[B47] Rodda S. J. McMahon A. P. (2006). Distinct roles for Hedgehog and canonical Wnt signaling in specification, differentiation and maintenance of osteoblast progenitors. *Development* 133 3231–3244. 10.1242/dev.02480 16854976

[B48] Schmitt-Ney M. (2020). The FOXO’s advantages of being a family: considerations on function and evolution. *Cells* 9:787. 10.3390/cells9030787 32214027 PMC7140813

[B49] Sweetser D. A. Peniket A. J. Haaland C. Blomberg A. A. Zhang Y. Zaidi S. T. (2005). Delineation of the minimal commonly deleted segment and identification of candidate tumor-suppressor genes in del(9q) acute myeloid leukemia. *Genes Chromosomes Cancer* 44 279–291. 10.1002/gcc.20236 16015647

[B50] Tang J. Xie J. Chen W. Tang C. Wu J. Wang Y. (2020). Runt-related transcription factor 1 is required for murine osteoblast differentiation and bone formation. *J. Biol. Chem.* 295 11669–11681. 10.1074/jbc.ra119.007896 32571873 PMC7450143

[B51] Velasco-Hernandez T. Sawen P. Bryder D. Cammenga J. (2016). Potential pitfalls of the Mx1-cre system: implications for experimental modeling of normal and malignant hematopoiesis. *Stem Cell Rep.* 7 11–18. 10.1016/j.stemcr.2016.06.002 27373927 PMC4945592

[B52] Visnjic D. Kalajzic Z. Rowe D. W. Katavic V. Lorenzo J. Aguila H. L. (2004). Hematopoiesis is severely altered in mice with an induced osteoblast deficiency. *Blood* 103 3258–3264. 10.1182/blood-2003-11-4011 14726388

[B53] Westendorf J. J. (2006). Transcriptional co-repressors of Runx2. *J. Cell. Biochem.* 98 54–64. 10.1002/jcb.20805 16440320

[B54] Wheat J. C. Krause D. S. Shin T. H. Chen X. Wang J. Ding D. (2014). The corepressor Tle4 is a novel regulator of murine hematopoiesis and bone development. *PLoS One* 9:e105557. 10.1371/journal.pone.0105557 25153823 PMC4143290

[B55] Wu M. Chen G. Li Y. P. (2016). TGF-β and BMP signaling in osteoblast, skeletal development, and bone formation, homeostasis and disease. *Bone Res.* 4:16009. 10.1038/boneres.2016.9 PMC4985055 27563484

[B56] Yin T. Li L. (2006). The stem cell niches in bone. *J. Clin. Invest.* 116 1195–1201. 16670760 10.1172/JCI28568 PMC1451221

[B57] Zhang P. Dressler G. R. (2013). The Groucho protein Grg4 suppresses Smad7 to activate BMP signaling. *Biochem. Biophys. Res. Commun.* 440 454–459. 10.1016/j.bbrc.2013.09.128 24099773 PMC3947529

[B58] Zhou X. Zhang Z. Feng J. Q. Dusevich V. M. Sinha K. Zhang H. (2010). Multiple functions of Osterix are required for bone growth and homeostasis in postnatal mice. *Proc. Natl. Acad. Sci. U.S.A.* 107 12919–12924. 10.1073/pnas.0912855107 20615976 PMC2919908

