# Supplementary material for: TLE4 Is a Critical Mediator of Osteoblast and Runx2-Dependent Bone Development
Source: Front Cell Dev Biol. 2021 Aug 6;9:671029. doi: 10.3389/fcell.2021.671029 (PMC8377417; doi:10.3389/fcell.2021.671029)
Supplement: Supplementary file 1 [file Data_Sheet_1.pdf]

Supplementary Material

1 Supplementary Table 1

Supplementary Table 1. List of mouse primers used for qRT-PCR (SYBR Green)

Gene	Forward (5' to 3')	Reverse (5' to 3')
Oc	ACAAGTCCCACACAGCAGCTT	GCCGGAGTCTGTTCACTACCT
Osx	GTACGGCAAGGCTTCGCATCTG	CTGATGTTTGCTCAAGTGGTCGC
Opg	GGCCTGATGTATGCCCTCAA	GTGCAGGAACCTCATGGTCTTC
Spp1	CCCGGTGAAAGTGACTGATTCT	GATCTGGGTGCAGGCTGTAAA
Ap	CACAGATTCCCAAAGCACCT	GGGATGGAGGAGAGAAGGTC
Runx2	GCCGGGAATGATGAGAACTA	GGTGAAACTCTTGCCTCGTC
18s	CGGAAATAGCCTTCGCCATCAC	ATCACTCGCTCCACCTCATCCT
Bmp2	ACTTTTCTCGTTTGTGGAGC	GAACCCAGGTGTCTCCAAGA
Bmp4	TGAGCCTTTCCAGCAAGTTT	CTTCCCGGTCTCAGGTATCA
Runx1	CACCGTCTTTACAAATCCGCCAC	CGCTCGGAAAAGGACAAACTCC

2 Supplementary Figure 1

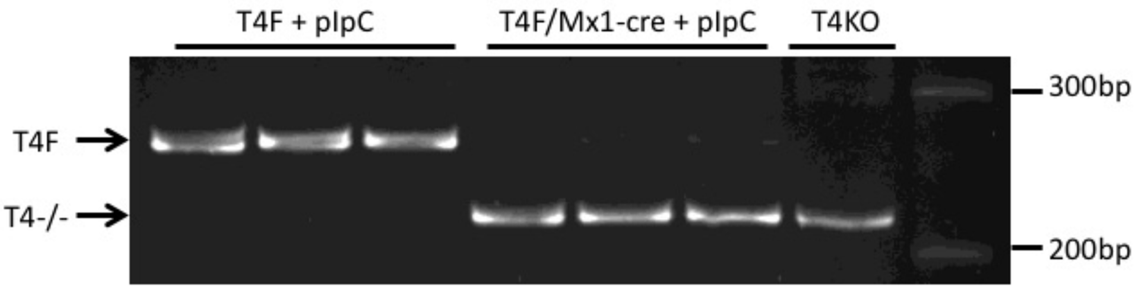

Supplementary Figure 1. High Efficiency of Tle4 knockout with Mx1-cre induction by pIpC in conditional Tle4 null mice. Mice homozygous for a conditional Tle4 allele consisting of *loxP*-flanked exon2 were treated with pIpC. Mice containing Mx1-cre demonstrate very high level of excision of exon 2 resulting in a reduced PCR product in this assay from 264 bp to 224 bp, matching the product seen in T4KO mice.

3. Supplementary Figure 2

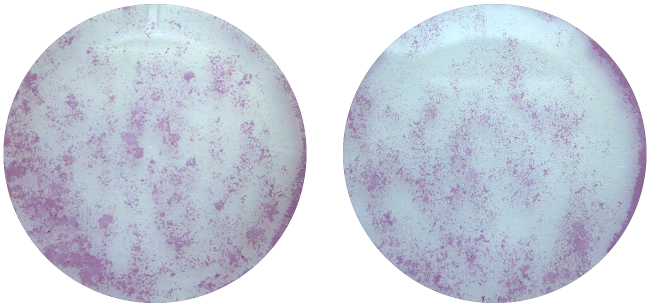

Supplementary Figure 2. Alkaline Phosphatase staining in stromal cells cultures generated from bones of one-week old T4WT or T4KO littermates.
